# Effects of Internet‐Based Lifestyle Interventions on Nonalcoholic Fatty Liver Disease: A Meta‐Analysis of Randomized Controlled Trials

**DOI:** 10.1155/cjgh/5125598

**Published:** 2026-04-21

**Authors:** Chengjie Wang, Yilong Song, Jingjing Wu, Hua Mei

**Affiliations:** ^1^ Shanghai University of Traditional Chinese Medicine Shanghai 201203 China shutcm.edu.cn; ^2^ Shanghai University of Medicine and Health Sciences Shanghai 201318 China sumhs.edu.cn

**Keywords:** internet-based intervention, lifestyle, meta-analysis, NAFLD

## Abstract

**Objective:**

This study aimed to evaluate the efficacy of internet‐based interventions in improving clinical outcomes among patients with nonalcoholic fatty liver disease (NAFLD) and to synthesize evidence for future research directions.

**Methods:**

A comprehensive search of nine electronic databases (CNKI, Wanfang, VIP, CBM, PubMed, Web of Science, Cochrane Library, Embase, and CINAHL) was conducted from inception to September 23, 2024, to identify randomized controlled trials (RCTs) assessing internet‐based lifestyle interventions in NAFLD patients. Two independent researchers screened studies, extracted data, and evaluated methodological quality. Meta‐analyses were performed using RevMan 5.3.5 software.

**Results:**

Ten RCTs involving 714 participants were included. Compared to the control group, the intervention groups showed significant reductions in BMI, ALT and AST. Weight loss trended toward reduction but was not statistically significant, though subgroup analysis showed significant weight loss in interventions lasting > 3 months.

**Conclusion:**

Internet‐based interventions significantly improve BMI and liver enzymes in NAFLD patients, with prolonged interventions (> 3 months) enhancing weight loss. Digital platforms show promise for enhancing self‐management, though standardized long‐term trials are needed to optimize efficacy.

## 1. Introduction

Over the past 2 decades, driven by energy‐dense, nutrient‐poor diets and sedentary lifestyles, global rates of obesity, Type 2 diabetes mellitus (T2DM), and metabolic syndrome (MetS) have steadily increased, leading to a parallel rise in nonalcoholic fatty liver disease (NAFLD) [1], which now affects approximately 32.4% of the global population [2]. Furthermore, the incidence of NAFLD is higher than in non‐China regions [3], and it has surpassed viral hepatitis to become the predominant liver disease in China.

NAFLD is a metabolic disorder primarily caused by overnutrition and insulin resistance (IR), resulting in hepatic steatosis without significant alcohol consumption [4]. The progression of NAFLD is strongly influenced by the capacity of adipose tissue and the liver to process excess nutrients [5]. If left unmanaged, NAFLD can progress from simple steatosis to steatohepatitis, fibrosis, and cirrhosis [6]. In addition, NAFLD is associated with extrahepatic complications, including cardiovascular disease and chronic kidney disease [7, 8], which significantly contribute to the clinical burden.

Lifestyle modification related to dietary habits and physical activities remains the cornerstone of NAFLD management [5]. Robust evidence indicates that a 7%–10% weight loss can improve metabolic disturbances and even reverse liver injury [9]. However, adherence to lifestyle changes is often poor, hindering effective treatment. A study revealed that adherence to the Mediterranean diet was low, with 58.6% of participants exhibiting poor compliance; alarmingly, the figure rose to 80.3% among those diagnosed with hepatic steatosis [10]. Therefore, sustainable interventions are urgently needed to provide the necessary support to help patients change their lifestyle more easily.

Internet‐based interventions have emerged as a promising solution for supporting lifestyle changes in patients with chronic conditions [11]. These digital platforms offer accessibility, convenience, and cost‐effectiveness, overcoming many of the barriers associated with traditional face‐to‐face healthcare delivery [12, 13]. Several new randomized controlled trials (RCTs) have been published. This study aims to synthesize the latest evidence to evaluate the efficacy of internet‐based lifestyle interventions on weight, body mass index (BMI), and liver enzymes in NAFLD patients, providing a contemporary evidence base to guide clinical practice and inform digital health integration.

## 2. Methods

This study adhered to the PRISMA guidelines [14], and the protocol was registered in PROSPERO (CRD420251234208).

### 2.1. Search Strategy

The search strategy was designed based on the principles of systematic literature retrieval outlined in the National Institute for Health and Care Excellence (NICE) guideline [15]. We conducted a systematic literature search of the CNKI, Wanfang, VIP, CBM, PubMed, Web of Science, Cochrane Library, Embase, and CINAHL databases on September 23, 2024. The search strategy included the following medical subject headings (MeSH) terms: “non‐alcoholic fatty liver disease,” “NAFLD,” “nonalcoholic fatty liver disease,” “nonalcoholic steatohepatitis,” “Internet‐based intervention,” “web‐based intervention,” “online intervention,” “telemedicine,” “mobile health,” and “telehealth.” Taking PubMed as an example, the search formula is presented in Table 1.

**TABLE 1:** An example of the search strategy in PubMed.

#1	Non‐alcoholic Fatty Liver Disease[MeSH Terms] OR Non alcoholic Fatty Liver Disease[Ti/Ab] OR Nonalcoholic Fatty Liver Disease[Ti/Ab] OR NAFLD[Ti/Ab] OR Nonalcoholic Fatty Liver[Ti/Ab] OR Nonalcoholic Steatohepatitis[Ti/Ab]
#2	Telemedicine[MeSH Terms] OR Mobile Health[Ti/Ab] OR mHealth[Ti/Ab] OR Telehealth[Ti/Ab] OR eHealth[Ti/Ab] OR Telecare[Ti/Ab]
#3	Internet‐based Intervention[MeSH Terms] OR Internet Intervention[Ti/Ab] OR Web‐based Intervention[Ti/Ab] OR Online Intervention[Ti/Ab]
#4	internet[Ti/Ab] OR online[Ti/Ab] OR website[Ti/Ab] OR computer[Ti/Ab] OR Wechat[Ti/Ab] OR electronic health[Ti/Ab]
#5	#2 OR #3 OR #4
#6	#1 AND #5

### 2.2. Eligibility Criteria

We considered the inclusion of literature in meta‐analyses if they met the following criteria: (1) the type of study was a RCT; (2) the subjects of the study were patients diagnosed with NAFLD by a reliable method, such as an imaging test; (3) the intervention group took web‐based interventions for lifestyle changes related to diet or physical activity through internet platforms such as apps and websites; and (4) the main outcome indicators were body weight and BMI. Secondary outcome measures were aspartate aminotransferase (AST) and alanine aminotransferase (ALT).

The exclusion criteria were as follows: (1) literature lacking original data or data with obvious errors; (2) the full text was unavailable; (3) duplicate publications; (4) animal experiments; (5) guidelines, expert consensus, and case reports; (6) review article and meta‐analysis; (7) the language of the literature is not Chinese or English; and (8) the outcome measures were inconsistent with this study.

### 2.3. Study Selection

The search results were imported into EndNote X8.2 for screening. Studies were screened and selected by 3 reviewers (Chengjie W, Yilong S, and Hua M). For the initial search, after removing duplicates, two researchers (Chengjie W and Yilong S) independently screened the titles and abstracts of the studies identified from 9 electronic databases based on the inclusion and exclusion criteria to eliminate irrelevant studies. In the second phase, the researchers (Chengjie W and Yilong S) reviewed the full texts of all remaining studies to determine the final set of included literature. Any disagreements encountered during the screening process by the 2 authors (Chengjie W and Yilong S) were resolved through discussion with a third researcher (Hua M).

### 2.4. Data Extraction

Two researchers (Chengjie W and Yilong S) extracted data and performed double‐checking independently. The following information was extracted from each selected study: first author, publication date, study location, study type, age of participants, sample size, intervention measures, intervention platform, intervention duration, and relevant outcomes. If some studies reported insufficient data for meta‐analysis, attempts were made to contact the original authors to obtain the missing data.

### 2.5. Data Synthesis and Statistical Analysis

Meta‐analysis was conducted using the Review Manager 5.3.5 and Stata Version 18.0. For all continuous outcome measures, the pooled effect size was expressed as the mean difference (MD) with 95% confidence intervals (CIs). Heterogeneity across the included studies was assessed using the *I* ^2^ statistic, with a significance level set at *p* < 0.10. A fixed‐effects model was employed if substantial heterogeneity was not present (*I* ^2^ < 50% and *p* > 0.10). Otherwise, a random‐effects model was used. Based on clinical experience, intervention duration was identified as a key potential effect modifier. The 3‐month cutoff for subgroup analysis was determined based on a combination of behavioral and physiological evidence. Behaviorally, the formation of healthy habits typically requires 2–5 months [16]. Physiologically, a previous study has validated 3 months as a reasonable period for observing short‐term effects, sufficient to detect significant changes in body weight and metabolic indicators [17]. Furthermore, this cutoff allowed for a balanced distribution of studies across subgroups. In cases of substantial heterogeneity (*I* ^2^ ≥ 50%), we conducted prespecified subgroup analyses (based on intervention duration) to explore potential sources. Sensitivity analyses were performed by sequentially excluding each study to assess the robustness of the pooled results. Publication bias was assessed for each outcome (body weight, BMI, ALT, and AST) using Egger’s linear regression test, implemented in Stata 18.0 with the meta‐analysis suite. A *p* value < 0.05 was considered indicative of statistically significant small‐study effects. Due to the limited number of studies in subgroup analyses (≤ 3 months vs. > 3 months), subgroup‐specific publication bias tests were not performed.

### 2.6. Risk of Bias Assessment

For included RCTs, the Cochrane Collaboration Risk of Bias tool was used to assess the quality of included studies. For RCTs, the evaluation criteria included the following: (1) random sequence generation, (2) allocation concealment, (3) blinding of participants and personnel, (4) blinding of outcome assessment, (5) incomplete outcome data, (6) selective reporting, and (7) other bias. Each domain was evaluated using the Cochrane risk ratings of “low,” “high,” and “unclear.” Two researchers (Chengjie W and Yilong S) independently completed the bias assessment, and any disagreement was resolved through discussion with a third researcher (Hua M).

## 3. Results

### 3.1. Study Selection

**FIGURE 1:**
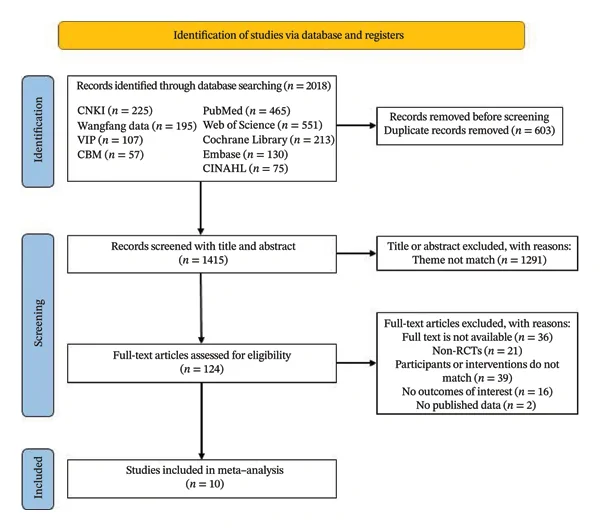
PRISMA 2020 flow diagram of study identification, screening, and inclusion.

A total of 2018 records were retrieved from 9 databases. After removing 603 duplicates, 1415 articles underwent title and abstract screening, leading to the exclusion of 1291 studies. We read the full text of 124 potentially relevant papers for further screening; ultimately, 10 of those met the inclusion criteria, with a total of 714 patients. The screening process is illustrated in Figure 1.

### 3.2. Characteristics of Included Studies

The included RCTs were published from 2018 to 2024 in 6 geographical regions (China, South Korea, Portugal, Australia, Singapore and the United States). The total sample size of the included studies was 714, the lowest number being 25, and the highest 108. The average age of the participants in the studies ranged from 18 to 78.72 years. All participants had NAFLD; however, Sara Policarpo [18] et al. recruited patients diagnosed with NAFLD who had HIV. Study characteristics are detailed in Table 2.

**TABLE 2:** Characteristics of the included studies (*n* = 10).

Author, year	Country	Study design	Study participants	Participants’ mean age (years)		Sample size (*n*)
				IG	CG	
Oh Young Kwon, 2024 [19]	South Korea	RCT	NAFLD patient	51.0 ± 13.37	47.1 ± 13.91	48/54

Eunbyul Cho, 2024 [20]	South Korea	RCT	NAFLD patient	41.32 ± 6.74	43.50 ± 6.67	25/24

Christine L. Freer, 2024 [21]	Australia	RCT	NAFLD patient	63.2 ± 7.7	63.9 ± 7.2	12/13

Jonathan G. Stine, 2023 [22]	United States	RCT	NASH patient	53.3 ± 13.3	50.4 ± 12.1	15/18

Sara Policarpo, 2021 [18]	Portugal	RCT	NAFLD patient with HIV	52.6 ± 9.9	55.9 ± 11.7	27/28

Su Lin Lim, 2020 [13]	Singapore	RCT	NAFLD patient	46.2 ± 11.0	46.1 ± 10.3	50/51

Meng, 2023 [23]	China	RCT	NAFLD patient	18–30:7	9	50/50
				31–45:20	20	
				46–59:23	21	

Zhao, 2019 [24]	China	RCT	NAFLD patient	65 ± 13		54/54

Yu, 2019 [25]	China	RCT	NAFLD patient	39.73 ± 16.6	40.67 ± 15.1	30/30

Huang, 2018 [26]	China	RCT	Old NAFLD patient	68.97 ± 8.98	69.71 ± 9.01	40/41

Abbreviations: CG, control group; HIV, human immunodeficiency virus; IG, intervention group; NASH, nonalcoholic steatohepatitis.

### 3.3. Intervention Characteristics

The characteristics of the interventions are presented in Table 3. Among the 10 included studies, the intervention groups received internet‐based lifestyle interventions where healthcare professionals provided medical advice on diet and exercise, along with online consultations and encouragement for adherence. In contrast, control groups mostly received usual standard care with traditional follow‐up methods (phone calls and text messages), while intervention groups utilized apps or video conferencing software. These include commercially available downloadable software and co‐developed apps. The duration of intervention ranged from 4 months to 2 years. In the included studies, we focused on changes in body weight, BMI, and liver enzymes (ALT and AST) as outcome measures of interest, with 2 studies [19, 20] specifically using the amount of the outcome change because their baselines are uneven.

**TABLE 3:** Characteristics of the interventions of included studies (*n* = 10).

Study	Interventions		Intervention platform	Intervention duration	Outcome
	IG	CG			
Kwon et al. [19]	Postinformation about diet and exercise Self‐monitoring: diet, caloric intake, etc. Online coaching: set weight loss goals, provide feedback and encouragement	Usual standard care	SMART‐Liver APP	6 months	①, ②, ③, ④

Cho et al. [20]	Condition evaluation Personalized nutritional prescriptions Self‐monitoring: weight, lifestyle, etc.	Usual standard care and educational materials	Dr.Coach APP	4 weeks	①, ②, ③, ④

Freer et al. [21]	Personalized exercise prescriptions Online consultation, healthy eating plan Provide incentives	Usual standard care	TeleHab, VALD Health, ZOOM	12 weeks	①

Stine et al. [22]	Educational articles Weight and diet records Physical activity counseling	Usual standard care	Noom Weight APP	16 weeks	①, ②, ③, ④

Policarpo et al. [18]	Structured diet Stimulates daily life and increases physical activity	General dietary recommendations	ZOOM	3 months	①, ②

Lim et al. [13]	Educational videos Food diary, setting goals (calorie intake, step count, etc.) Peer chat, weight recording, and reminder punch‐in	Usual standard care	nBuddy APP	6 months	①, ②, ③, ④

Meng et al. [23]	WeChat‐based multidisciplinary collaborative health management, including nutrition intervention, exercise intervention, etc.	Patient self‐management	WeChat	6 months	①, ③, ④

Zhao et al. [24]	Personalized health intervention program, correction of unhealthy lifestyle, regular follow‐up, etc.	Usual standard care	Health management system platform (jointly developed with Hangzhou Xihe Information Technology Co., Ltd.)	2 years	②, ③, ④

Yu et al. [25]	WeChat: consultation and professional knowledge Mint management APP: measure BMI, calculate food calories, etc. Codoon APP: set schedules, calculate consumption, etc.	Routine medical treatment, diet and exercise follow‐up	WeChat, Mint management APP, Codoon APP	3 months	②

Huang et al. [26]	Electronic device management: set tasks in the mobile app, and send intervention reminders if they are not completed	Face‐to‐face counseling and guidance, regular text or phone follow‐up	APP	3 months	①, ②, ③, ④

*Note:* Outcome: ① weight (kg); ② BMI; ③ ALT (IU/L); ④ AST (IU/L).

### 3.4. Meta‐Analysis of Weight, BMI, ALT, and AST

#### 3.4.1. Weight

**FIGURE 2:**
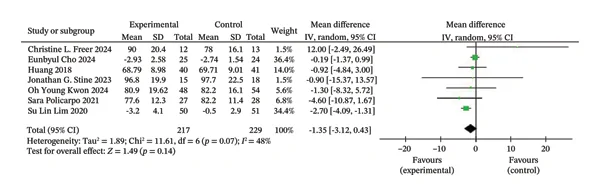
Forest plot of body weight change in NAFLD patients receiving internet‐based lifestyle interventions vs. control groups. The pooled analysis showed no statistically significant reduction in body weight overall (MD = −1.35 kg, 95% CI: −3.12 to 0.43, *p* = 0.14). Mild heterogeneity was observed (*I* ^2^ = 48%, *p* = 0.07). CI: confidence interval; MD: mean difference.

**FIGURE 3:**
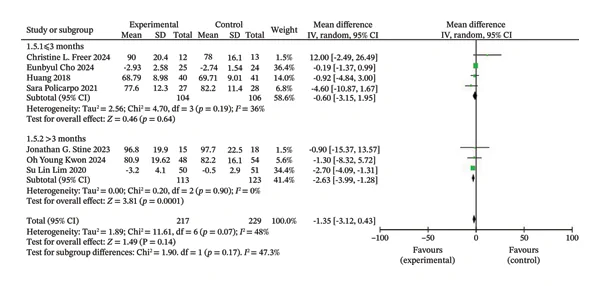
Subgroup analysis of body weight change stratified by intervention duration (> 3 months vs. ≤ 3 months). Interventions lasting > 3 months resulted in a significant reduction in body weight (MD = −2.63 kg, 95% CI: −3.99 to −1.28, *p* = 0.0001), whereas shorter interventions (≤ 3 months) did not demonstrate a significant effect (MD = −0.60 kg, *p* = 0.64).

All selected studies were included in the meta‐analysis. Seven studies chose body weight as the outcome indicator. There was mild heterogeneity among the studies (*p* = 0.07, *I* ^2^ = 48%). The random‐effects model showed that compared to conventional care, internet‐based lifestyle intervention did not significantly reduce the body weight of NAFLD patients (MD = −1.35, 95% CI: [−3.12, 0.43], *Z* = 1.49, *p* = 0.14), as shown in Figure 2. Subgroup analysis showed that an intervention duration of more than 3 months could significantly reduce the body weight of NAFLD patients (MD = −2.63, 95% CI: [−3.99, −1.28], *Z* = 3.81, *p* = 0.0001), and no heterogeneity was found (*I* ^2^ = 0%, *p* = 0.90); an intervention duration of ≤ 3 months had no obvious effect on reducing the body weight of NAFLD patients (MD = −0.60, 95% CI: [−3.15, 1.95], *Z* = 0.46, *p* = 0.64), and no heterogeneity was found (*I* ^2^ = 36%, *p* = 0.19) (Figure 3).

#### 3.4.2. BMI

**FIGURE 4:**
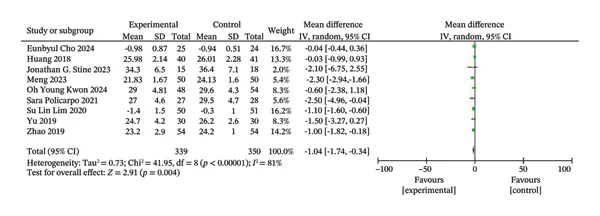
Forest plot of BMI change in NAFLD patients receiving internet‐based lifestyle interventions vs. control groups. The intervention group showed a statistically significant reduction in BMI compared to the control group (MD = −1.04 kg/m^2^, 95% CI: −1.74 to −0.34, *p* = 0.004). Significant heterogeneity was observed among the studies (*I* ^2^ = 81%, *p* < 0.00001).

**FIGURE 5:**
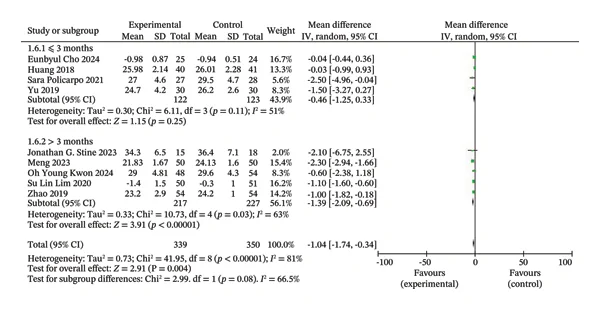
Subgroup analysis of BMI change stratified by intervention duration (> 3 months vs. ≤ 3 months). A significant improvement in BMI was observed in the group with an intervention duration of > 3 months (MD = −1.39 kg/m^2^, 95% CI: −2.09 to −0.69, *p* < 0.0001), while the ≤ 3 months group showed no significant change (*p* = 0.25).

Nine studies reported the BMI outcomes of NAFLD patients. There was significant heterogeneity among the studies (*p* < 0.00001, *I* ^2^ = 81%), and a random‐effects model was used for meta‐analysis, as shown in Figure 4. The results showed that the BMI of the intervention group was lower than that of the control group, and the difference was statistically significant (MD = −1.04, 95% CI: [−1.74, −0.34], *Z* = 2.91, *p* = 0.004). Subgroup analysis was conducted to explore the source of heterogeneity. The results showed that the BMI of the group with an intervention duration of more than 3 months was significantly improved (MD = −1.39, 95% CI: [−2.09, −0.69], *Z* = 3.91, *p* < 0.0001), while the BMI of the group with an intervention duration of ≤ 3 months did not change significantly (MD = −0.46, 95% CI: [−1.25, 0.33], *Z* = 1.15, *p* = 0.25) (Figure 5).

#### 3.4.3. ALT

**FIGURE 6:**
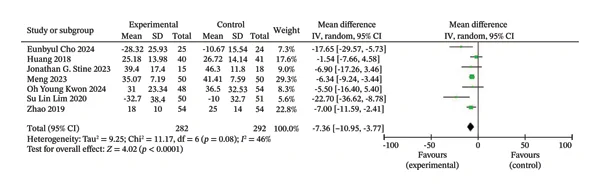
Forest plot of ALT change in NAFLD patients receiving internet‐based lifestyle interventions versus control groups. The internet‐based intervention significantly reduced ALT levels compared to standard care (MD = −7.36 IU/L, 95% CI: −10.95 to −3.77, *p* < 0.0001). Mild heterogeneity was present (*I* ^2^ = 46%, *p* = 0.08).

**FIGURE 7:**
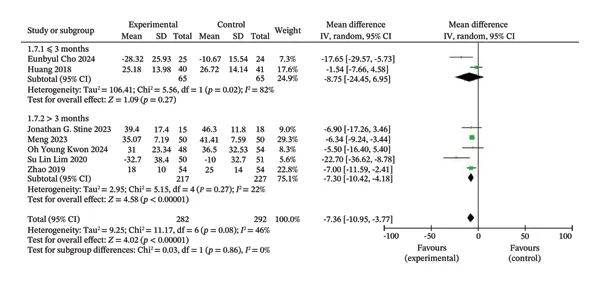
Subgroup analysis of ALT change stratified by intervention duration (> 3 months vs. ≤ 3 months). Interventions lasting > 3 months significantly reduced ALT levels (MD = −7.30 IU/L, 95% CI: −10.42 to −4.18, *p* < 0.0001), whereas interventions ≤ 3 months did not show a significant reduction (*p* = 0.27).

Seven studies reported the changes in ALT levels of NAFLD patients. There was mild heterogeneity among the studies (*p=*0.08, *I* ^2^ = 46%), and a random‐effects model was used for meta‐analysis. The results showed that the ALT values in the intervention group were significantly lower than those in the control group, with statistically significant differences (MD = −7.36, 95% CI: [−10.95, −3.77], *Z* = 4.02, *p* < 0.0001), as shown in Figure 6. Subgroup analysis showed that an intervention duration of more than 3 months could significantly reduce the ALT levels in NAFLD patients (MD = −7.30, 95% CI: [−10.42, −4.18], *Z* = 4.58, *p* < 0.00001), and no heterogeneity was found (*I* ^2^ = 22%, *p* = 0.27); an intervention duration of ≤ 3 months had no obvious effect on reducing the ALT levels in NAFLD patients (MD = −8.75, 95% CI: [−24.45, 6.95], *Z* = 1.09, *p* = 0.27) (Figure 7).

#### 3.4.4. AST

**FIGURE 8:**
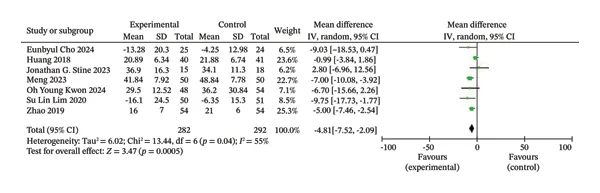
Forest plot of AST change in NAFLD patients receiving internet‐based lifestyle interventions vs. control groups. The pooled effect indicated a significant reduction in AST levels in the intervention group (MD = −4.81 IU/L, 95% CI: −7.52 to −2.09, *p* = 0.0005). Moderate heterogeneity was observed (*I* ^2^ = 55%, *p* = 0.04).

**FIGURE 9:**
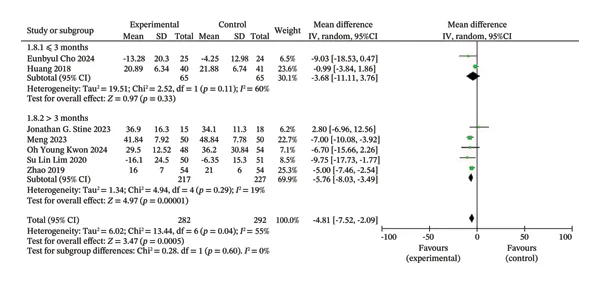
Subgroup analysis of AST change stratified by intervention duration (> 3 months vs ≤ 3 months). Subgroup analysis revealed that interventions lasting > 3 months significantly improved AST levels (MD = −5.76 IU/L, 95% CI: −8.03 to −3.49, *p* < 0.0001), while the effect was not significant for interventions ≤ 3 months (*p* = 0.33).

Seven studies selected AST as the outcome indicator, and there was mild heterogeneity among the studies (*p* = 0.04, *I* ^2^ = 55%). A random‐effects model was used for meta‐analysis, and the results showed that the combined effect was statistically significant (MD = −4.81, 95% CI: [−7.52, −2.09], *Z* = 3.47, *p* = 0.0005), as shown in Figure 8. Subgroup analysis showed that compared to the control group, internet‐based lifestyle intervention for more than 3 months could significantly improve AST in NAFLD patients (MD = −5.76, 95% CI: [−8.03, −3.49], *Z* = 4.97, *p* < 0.00001), while the combined effect of ≤ 3 months was not statistically significant (MD = −3.68, 95% CI: [−11.11, 3.76], *Z* = 0.97, *p* = 0.33) (Figure 9).

#### 3.4.5. Publication Bias

Egger’s linear regression test was performed to assess publication bias for each primary outcome. No significant small‐study effects were detected for body weight (*p* = 0.180), BMI (*p* = 0.726), ALT (*p* = 0.777), or AST (*p* = 0.268). Test result charts for each outcome are presented in Supporting Information (Publication bias).

#### 3.4.6. Risk of Bias

**FIGURE 10:**
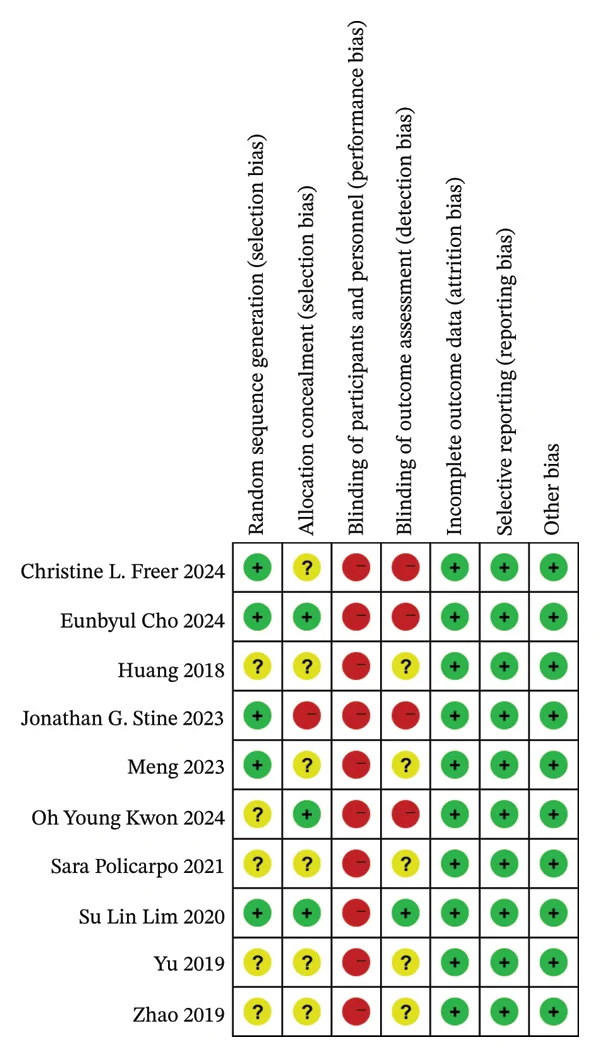
Risk of bias assessment of the 10 included randomized controlled trials using the Cochrane Collaboration Risk of Bias tool. Green (+) = low risk; yellow (?) = unclear risk; red (−) = high risk.

The risk of bias of the 10 studies included in this study was assessed in Figure 10. Overall, the risk of bias reported in the studies was low for incomplete outcome data, selective reporting, and other bias. Due to the nature of the RCTs, it was difficult to blind participants in all studies, resulting in a high risk of bias. In the blinding of outcome assessment, only one article [13] did it. In addition, only three studies performed allocation concealment, and most studies were unclear on this topic. Half of the studies were at low risk of selection bias, and the other half were unclear.

## 4. Discussion

Our meta‐analysis of 10 RCTs involving 714 NAFLD patients demonstrates that internet‐based lifestyle interventions can significantly improve patients’ BMI, ALT, and AST levels. Although the overall weight loss did not reach statistical significance (MD = −1.35 kg, *p* = 0.14), subgroup analysis revealed a key finding: when the intervention duration exceeded 3 months, patients experienced significant weight loss (MD = −2.63 kg, *p* = 0.0001), and further improvements in BMI and liver enzymes were observed.

Previous systematic reviews and meta‐analyses have consistently confirmed that remote interventions significantly reduce ALT and AST levels in NAFLD patients with NAFLD [27–30], which aligns with our findings. Notably, Saokaew et al. [27] reported the greatest reduction in liver enzymes, a finding that may be attributed to the inclusion of obese NAFLD patients, whose higher baseline levels provided greater room for improvement postintervention. Evidence regarding body weight remains divergent. While Kwon and Saokaew’s studies did not report weight changes [27, 29], Kumar’s study [28] demonstrated significant weight loss, contrasting with our overall findings. In their systematic review, Seifi observed that six out of seven studies reported significant weight reduction, leading to their conclusion that electronic interventions are mostly effective [30]. However, this narrative summary masks substantial interstudy heterogeneity. Our subgroup analysis revealed that significant weight loss was achieved only when interventions exceeded 3 months in duration. The positive findings reported by Kumar [28] can be explained by their longer intervention period (≥ 16 weeks), which aligns with our subgroup results. Regarding BMI improvement, our findings are consistent with those of Kwon [29] but diverge from Kumar and Saokaew [27, 28]. Seifi also noted that five out of six studies reporting BMI showed significant reductions [30], consistent with our overall positive results. However, they emphasized that the heterogeneity of intervention modalities (telephone, text messaging, and mobile applications) makes it difficult to determine the optimal approach [30]. We argue that this heterogeneity can explain the discrepancies across studies. On one hand, the nature of the intervention modality matters: Kumar [28] relied on unidirectional electronic interventions (such as text message reminders), whereas the studies included in our meta‐analysis predominantly employed interactive applications and websites, which more effectively facilitated behavioral change. On the other hand, population characteristics cannot be overlooked: the obese NAFLD patient likely required more intensive and prolonged interventions, and BMI may be less sensitive to short‐term changes compared to liver enzymes in this population [27].

Lifestyle changes, such as dietary modifications and increased physical activity, are the cornerstone of NAFLD management [4, 5]. The pathogenesis of NAFLD is primarily driven by IR, oxidative stress, and hepatic lipid accumulation [31], with weight loss serving as the key to breaking this vicious cycle. However, traditional offline interventions often face challenges such as limited patient time and energy, insufficient disease awareness, and lack of feedback, resulting in poor adherence [32]. With the widespread use of mobile devices, internet‐based lifestyle interventions can overcome temporal and spatial barriers, continuously collect data, educate, and supervise patients with chronic diseases, thereby improving disease outcomes [11].

Internet‐based lifestyle interventions represent not only a change in medium but a reconstruction of the intervention model. Traditional interventions rely primarily on patient recall and clinic‐based monitoring, which are often compromised by memory bias and lagged feedback. In contrast, digital interventions facilitate immediate feedback loops, allowing patients to record metrics such as dietary intake and physical activity in real‐time [13, 19]. These data are then transformed into visual analytics. Some advanced programs even integrate artificial intelligence to provide instantaneous caloric analysis and nutritional counseling based on photographs of meals [13]. Such visibility allows patients to intuitively monitor their progress and identify setbacks, thereby strengthening coping appraisals and correcting maladaptive behaviors [33]. Furthermore, internet‐based platforms utilize gamification elements, such as points and leaderboards, to provide instantaneous rewards. By stimulating dopamine secretion, these features counteract the perceived monotony of maintaining healthy behaviors. This neurobiological feedback encourages the consolidation of repetitive actions into lasting habits, significantly enhancing long‐term adherence [34]. Improved adherence not only sustains behavioral change but also translates into physiological benefits over time, including enhanced insulin sensitivity and reduced adipose accumulation [35], ultimately leading to statistically significant improvements in body weight and liver enzyme profiles [36]. However, as longitudinal interventions require substantial time and resources, patient attrition remains a challenge; thus, adherence remains a critical metric in evaluating intervention efficacy. Crucially, internet‐based interventions foster the creation of online communities and peer support networks [33]. Patients often perceive experiential sharing from peers in similar health conditions as more egalitarian and emotionally resonant than professional clinical guidance. This robust emotional support is highly effective in alleviating anxiety and bolstering self‐efficacy [37], which are essential drivers for the sustained maintenance of behavioral changes.

Our findings have important clinical implications. First, clinical guidelines indicate that in the early stages of NAFLD development, a 5%–10% weight loss can reverse the disease [4]. However, due to limited medical resources, patients often lack specific doctor guidance during visits, while internet‐based interventions can set weight loss goals for patients and break them down into daily executable diet and exercise tasks, helping to improve IR and prevent progression from simple steatosis to the inflammatory stage. When the disease progresses to NASH, patients often have no obvious symptoms, but hepatocyte damage causes elevated ALT and AST levels; at this point, internet‐based interventions can regularly push review reminders for timely intervention, preventing patients from unknowingly progressing from inflammation to liver fibrosis, as fibrosis is the final gateway to cirrhosis and liver cancer [38, 39]. Second, our data emphasize the importance of sustained engagement in digital therapies: clinicians should inform patients that at least 3 months of adherence are necessary to achieve significant weight loss effects. Although long‐term interventions seem to produce better results, it is important to note that short‐term interventions may still offer some benefits [40, 41]. Studies have shown that even modest weight loss can provide metabolic benefits and can be used in conjunction with other treatment strategies. In addition, telemedicine technologies have fundamentally overcome geographical barriers, eliminating reliance on physical clinics. As long as mobile network coverage is available, standardized interventions can reach patients in remote areas at a negligible cost. The scalability of digital platforms makes them an ideal tool for maintaining continuity of NAFLD care in resource‐limited settings (or during events such as the COVID‐19 pandemic [18]).

Our study has several limitations. First, due to the varied forms of internet interventions, which adopted diverse application software (such as social media and video conferencing software), the included studies exhibited high heterogeneity. However, this also reflects the flexibility of internet medical approaches; compared to traditional phone or text message reminders, using apps and websites typically provides stronger behavior change support, leading to better clinical outcomes. Future research needs to further standardize intervention protocols to determine the most cost‐effective intervention combinations. Moreover, intervention duration is closely related to efficacy; our subgroup analysis confirmed it as a key moderating factor. Although short‐term interventions may yield health benefits on certain metabolic indicators, to ensure persistence, we recommend a minimum intervention duration of 3 months. The shortest follow‐up in current studies is only 4 weeks, so the effectiveness of internet interventions and patient adherence still requires more long‐term follow‐up data for confirmation. Second, due to the nature of lifestyle interventions, it was not possible to blind participants, which introduced a risk of implementation bias. Some studies also had unclear allocation concealment. To address this, we conducted sensitivity analyses by excluding each study individually and confirmed the stability of the pooled effect size. Furthermore, internet‐based interventions are limited by the accessibility of devices and networks, which may introduce selection bias. We included additional studies from developing regions by searching Chinese databases (such as CNKI), which somewhat enhanced the generalizability of our results. Finally, our study relied on surrogate markers (weight, BMI, ALT, and AST) consistent with previous meta‐analyses, because many original studies did not report imaging or histological outcomes. While these biochemical and anthropometric indicators are widely accepted as representative endpoints reflecting improvements in metabolic health and hepatic inflammation, their use may restrict the ability to definitively infer the long‐term efficacy of internet‐based interventions on histological regression. Consequently, the lack of histological evidence, including results from FibroScan or MRI, means that the long‐term impact of internet‐based interventions on preventing progression to cirrhosis has yet to be fully elucidated.

The strengths of our study include strict adherence to PRISMA guidelines and registration; the exclusion of studies using phone‐based electronic health interventions in other meta‐analyses, focusing on internet‐based interventions; and the inclusion of studies from Chinese databases, offering a more comprehensive search. Future RCTs should standardize intervention protocols and durations and include more comprehensive outcome measures to confirm efficacy.

## 5. Conclusion

Internet‐based lifestyle interventions demonstrate benefits in reducing weight, BMI, and liver enzymes in NAFLD patients, particularly with interventions lasting > 3 months. These findings support the integration of digital tools into NAFLD care pathways. Future research should focus on optimizing intervention duration, intensity, and scalability through multicenter trials to establish long‐term efficacy and cost‐effectiveness.

## Author Contributions

Hua Mei developed the research question and reviewed the final draft. Chengjie Wang and Yilong Song participated in screening, extraction, and quality assessment. Chengjie Wang and Jingjing Wu analyzed the data, performed data tabulation, interpreted the results, and drafted the manuscript.

## Funding

The authors received no specific funding for this work.

## Disclosure

All authors have read and contributed to the final manuscript.

## Ethics Statement

This study did not involve human participants or animal experiments and therefore required no ethical review approval.

## Conflicts of Interest

The authors declare no conflicts of interest.

## Supporting Information

Additional supporting information can be found online in the Supporting Information section.

## Supporting information


**Supporting Information 1** PRISMA 2020 checklist.

**Supporting Information 2** Publication bias.

## Data Availability

The data that support the findings of this study are available from the corresponding author upon reasonable request.
