# Correction to “Effects of Internet‐Based Lifestyle Interventions on Nonalcoholic Fatty Liver Disease: A Meta‐Analysis of Randomized Controlled Trials”

**DOI:** 10.1155/cjgh/9837697

**Published:** 2026-11-09

C. Wang, Y. Song, J. Wu, H. Mei, Effects of Internet‐Based Lifestyle Interventions on Nonalcoholic Fatty Liver Disease: A Meta‐Analysis of Randomized Controlled Trials, *Canadian Journal of Gastroenterology and Hepatology*, 2026, 5125598, 12 pages, 2026, https://doi.org/10.1155/cjgh/5125598.

In the author affiliation information, the corresponding author’s affiliation was published incorrectly. The published version listed only:

^2^Shanghai University of Medicine and Health Sciences, Shanghai 201318, China

The correct affiliation information for the corresponding author should be:

^1^Shanghai University of Traditional Chinese Medicine, Shanghai 201203, China

^2^Shanghai University of Medicine and Health Sciences, Shanghai 201318, China

The affiliation listing should be corrected accordingly to include both institutions as shown above.

We apologize for this error.

