# Supplementary material for: Effects of Internet‐Based Lifestyle Interventions on Nonalcoholic Fatty Liver Disease: A Meta‐Analysis of Randomized Controlled Trials
Source: Can J Gastroenterol Hepatol. 2026 Apr 21;2026:5125598. doi: 10.1155/cjgh/5125598 (PMC13580839; doi:10.1155/cjgh/5125598)
Supplement: Supplementary file 1 [file CJGH-2026-5125598-s002.pdf]

Weight

Number of studies = 7 Root MSE = 1.328

Std_Eff	Coefficient	Std. err.	t	P>|t|	[95% conf.	interval]
slope	-1.048866	.5425939	-1.93	0.111	-2.443648	.3459164
bias	3.338616	2.140254	1.56	0.180	-2.163083	8.840315

Test of H0: no small-study effects P = 0.180

BMI

Number of studies = 9 Root MSE = 2.069

Std_Eff	Coefficient	Std. err.	t	P>|t|	[95% conf.	interval]
slope	-.829141	.9377732	-0.88	0.406	-3.046622	1.38834
bias	1.455454	3.992427	0.36	0.726	-7.985136	10.89604

Test of H0: no small-study effects P = 0.726

ALT

Number of studies = 7 Root MSE = 1.413

Std_Eff	Coefficient	Std. err.	t	P>|t|	[95% conf.	interval]
slope	-.3196521	.6413222	-0.50	0.639	-1.968223	1.328919
bias	-.8513129	2.842152	-0.30	0.777	-8.157298	6.454672

Test of H0: no small-study effects P = 0.777

AST

Number of studies = 7 Root MSE = 1.405

Std_Eff	Coefficient	Std. err.	t	P>|t|	[95% conf.	interval]
slope	-1.289919	.6615081	-1.95	0.109	-2.99038	.4105417
bias	3.657921	2.93345	1.25	0.268	-3.882752	11.19859

Test of H0: no small-study effects P = 0.268
